# Profiles of Women With Fibromyalgia and Social Comparison Processes

**DOI:** 10.3389/fpsyg.2020.00440

**Published:** 2020-03-13

**Authors:** M. Carmen Terol Cantero, Abraham P. Buunk, Victor Cabrera, Miguel Bernabé, Maite Martin-Aragón Gelabert

**Affiliations:** ^1^Departamento Ciencias del Comportamiento y Salud, Facultad Ciencias Sociosanitarias, Universidad Miguel Hernández, Elche, Spain; ^2^Royal Netherlands Academy of Arts and Sciences (KNAW), Amsterdam, Netherlands; ^3^Departamento de Psicología de la Salud, Universidad Miguel Hernández, Elche, Spain; ^4^Departamento de Psicología Social y de las Organizaciones, Facultad de Psicología, Universidad Nacional de Educación a Distancia, Madrid, Spain

**Keywords:** social comparison, fibromyalgia, patient profiles, anxiety, depression

## Abstract

**Background:**

Due to uncertainty regarding chronic pain in Fibromyalgia (FM) patients, there has been a growing interest in social comparison and its influence on emotional responses.

**Aims:**

to analyze profiles in FM patients according to pain perception, social comparison strategies and anxiety and depression.

**Methods:**

The sample consisted of 131 FM outpatients (Mean age: 50.15, *SD* = 11.1). Two scales were used: the Social Comparison Illness Scale and the Hospital Anxiety and Depression Scale.

**Results:**

Two profiles were found by cluster analysis (K-means method): one (66%) with a higher level of *pain perception, anxiety* and *depression* and greater use of *upward contrast* and *downward identification* social comparison; and another (34%) with lower levels of *pain perception, anxiety* and *depression* and greater use of *upward identification* and *downward contrast*.

**Conclusion:**

These profiles underline the interest in social comparison strategies and their role in FM.

## Introduction

Fibromyalgia (FM) is a chronic disease that mainly affects women and is characterized by widespread musculoskeletal pain accompanied by various symptoms such as fatigue, stiffness, sleep disruption, physical symptoms (i.e., extreme sensitivity, headaches, irritable bowel syndrome, temporomandibular joint disorders) and high levels of anxiety and depression (Wolfe et al., 2010, 2013). The heterogeneity of these symptoms is one of the reasons why researchers have tried to analyze different patient profiles and their relationship with psychological adaptation. They present a “maladaptive profile” with higher levels of *pain perception*, *anxiety* and *depression*, in contrast to an “adaptive profile” with moderate/low levels of *pain perception*, *anxiety* and *depression* (Kurtze et al., 1998; Giesecke et al., 2003; Shuster et al., 2009; Calandre et al., 2011; Keller et al., 2011; Docampo et al., 2013). *Anxiety* and *Depression* could be important indicators for predicting a patient profile with a worse prognosis, more severe symptoms, pain perception and fewer functional abilities (Bennett, 2002; Thieme et al., 2004; De Souza et al., 2009; Calandre et al., 2011). However, cognitive processes are seen to have a fundamental role in reducing or dealing with anxiety and depression symptoms in FM (Rodero et al., 2010; Montesó-Curto et al., 2015; Peñacoba-Puente et al., 2015; Cabrera-Perona et al., 2017; Pastor-Mira et al., 2017).

According to the social comparison theory, lack of information and uncertainty can trigger cognitive processes of social comparison (Festinger, 1954). Indeed, chronic patients with higher uncertainty show more anxiety and depression symptoms and interest in social comparison (Butzer and Kuiper, 2006; Terol et al., 2007b, 2012, 2014; Terol-Cantero et al., 2015; Cabrera-Perona et al., 2017). These patients usually compare themselves with “others” or “*referents”* who are considered psychologically close or in a similar situation (e.g., same diagnosis) (Suls et al., 2002; Buunk and Gibbons, 2006; Corcoran et al., 2011). They compare “*contents”* such as symptoms, ways of coping or adjustment to chronic pain or illness (Butzer and Kuiper, 2006; Dibb and Yardley, 2006; Mussweiler et al., 2006; Jauregui-Lobera et al., 2010; Corcoran et al., 2011).

More specifically, the Identification-Contrast Model (Buunk and Ybema, 1997; Buunk and Gibbons, 2007) suggests that social comparison with “*referents*” either focusing on similarities with “others” who are better-off (*upward identification*), or focusing on contrast with “*referents*” who are worse-off (*downward contrast*) would create a positive affect (Buunk et al., 1990; Smith, 2000). However, social comparisons with better-off “others” while focusing on differences (*upward contrast*), or with worse-off “others” while perceiving similarities (*downward identification*) would lead to negative affect (Buunk et al., 1990; Smith, 2000). In chronic illness or pain, social comparison *“strategies*” such as *upward identification* and *downward contrast* have been associated with lower depression and better psychosocial adjustment (Van der Zee et al., 1996, 2000; Terol et al., 2012); and *upward contrast* or *downward identification* have been linked to higher depression and worse adjustment (Neugebauer et al., 2003; Terol et al., 2007b, 2014). In the same way, a few studies on FM have shown that *upward identification* or *downward contrast* strategies are related to lower pain perception and better mood (Affleck et al., 2000; Terol et al., 2014; Cabrera-Perona et al., 2017) and *upward contrast* or *downward identification* are associated with higher levels of anxiety and depression, and worse psychological adjustment (Affleck et al., 2000; Groothof and Scholtes, 2007; Terol et al., 2014; Cabrera-Perona et al., 2017).

In the context of the above, the aim of this study was to analyze the profiles of women with FM who share common characteristics based on a set of assessed variables: *pain perception*, *social comparison* processes (*strategies*, *referents*, and *contents*) and *anxiety* and *depression*.

## Materials and Methods

### Sample

The sample consisted of 131 Spanish female outpatients interviewed at San Vicente del Raspeig Hospital (FM Department). The mean age was 50.15 (*SD* = 11.14). Mean time since diagnosis was 4.32 years (*SD* = 4.99). 68.70% of the participants were married and 31.3% were single, separated-divorced or widows. Educational level was primary and secondary school (77%), higher education (10.7%), and read/write (12.3%). Inclusion criteria were: (1) FM diagnosis re-confirmed by the American College of Rheumatology (ACR) criteria (Wolfe et al., 2010) upon their arrival at the FM Department, (2) aged over 18, (3) no previous psychiatric diagnosis (4) ability to understand questionnaires, (5) informed consent to participate in the study.

### Assessments

In addition to collecting information about age, marital status, educational level and time since diagnosis, the following scales were used to assess the variables used in the study:

*Pain perception Visual Analog Scale* (VAS: Aliaga-Font, 2009) was used to assess: current pain, average pain last week, and maximum pain last week. Patients had to mark their pain perception for each of the three times on the VAS (0 = no pain to 10 = worst imaginable pain).

*Social Comparison Process in Illness scale* (adapted from Van der Zee et al. (2000) by Terol et al. (2007a, 2014). This scale includes 18-ítems with a Likert response-scale (1 = never; 5 = very often) grouped into three subscales: Social Comparison Strategies, Social Comparison Referents, and Social Comparison Contents. Three items are included in each of the four Social comparison strategies: *upward identification (a = 0.89), upward contrast (a = 0.84), downward identification (a = 0.93), and downward contrast* (*a* = 0.75). The *referents* subscale includes three items (“others” with similar health problems, with different health problems, and with no health problems) and *contents* also includes three items (symptoms, mood, and physical activity). Higher scores show a greater frequency in patients’ use of social comparison strategies, referents or contents.

*Hospital Anxiety and Depression Scale* (HADS: Zigmond and Snaith, 1983; Spanish adaptation by Terol et al., 2007a). This is a 14-item scale consisting of two 7-item subscales: Anxiety and Depression. Responses are given on Likert scales from 0 to 3 with a 0–21 range for each subscale. Higher scores show higher levels of anxiety and/or depression. Internal consistency for this study, HADS – Anxiety α = 0.80, HADS – Depression α = 0.85.

### Procedure

This was a cross-sectional study with a non-probability convenience sample. After the Hospital Ethics Committee’s approval of the study, we selected 152 newly admitted outpatients with FM diagnosis. Patients were informed of the study and they signed an informed consent. 13 of these patients refused to participate and eight did not meet the inclusion criteria. Subsequently, 131 outpatients were interviewed by a psychologist in sessions lasting from 20 to 30 min.

### Statistical Analysis

The software IBM SPSS v.22 was used for the statistical analysis, and the Kolmogorov–Smirnov test was carried out for distribution of scores (HADS: *D* = 0.057; *p* = 0.20; Social Comparison Processes in Illness Scale: *D* = 1.24; *p* = 0.000). Means and frequencies were used for the *Descriptive Analyses*. For *Patients’ Profile*s, an iterative K-means cluster analysis (non-hierarchical method) was performed to identify subgroups (*K* = 2) and differences were analyzed by ANOVA (F-Fisher with *p* < 0.05 were accepted). Prior to clustering, multicollinearity was assessed (VIFs < 6). Contingency tables and *χ*^2^ statistics were used for the sample distribution “case” / “non-case” according to the HADS and inclusion in either of the profiles. In FM, specific cut-off points for those considered “cases” were recently fixed at +12 for the HADS-Anxiety and HADS-Depression subscales (see Cabrera et al., 2015).

## Results

### Descriptive Analysis

Table 1 shows means, standard deviations and range scores for all study variables. Frequency in patient’s use of Social Comparison strategies referents or contents are presented in Table 2.

**TABLE 1:** Descriptive analysis: Means, standard deviations, and range scores for all variables.

	Total sample = 131		
	*M*	*SD*	Range
*Pain perception Visual Analog Scale*			
Current pain	5.48	1.56	0–10
Last week average pain	6.58	1.56	0–10
Maximum pain last week	7.18	1.44	0–10
*Social Comparison Processes in Illness Scale Strategies*			
Upward identification	9.31	2.97	3–15
Upward contrast	11.30	3.11	3–15
Downward identification	9.98	3.63	3–15
Downward contrast	9.10	3.01	3–15
*Referents*			
Similar health problems	3.37	1.35	1–5
Different health problems	2.98	1.24	1–5
No health problems	3.02	1.49	1–5
*Contents*			
Symptoms	4.36	.81	1–5
Mood	3.89	1.05	1–5
Physical activity	4.02	1.22	1–5
*Hospital Anxiety and Depression Scale*			
Anxiety	13.71	4	0–21
Depression	10.73	4.64	0–21

*M, mean; SD, Standard Deviation.*

**TABLE 2:** Descriptive analysis: Frequency in patient’s use of social comparison strategies referents or contents according to three categories (*).

	Total sample = 131		
	Low	Medium	High
	frequency	frequency	frequency
*Social Comparison Strategies*			
Upward identification	18.3%	39.7%	42%
Upward contrast	9.9%	14.5%	75.6%
Downward identification	19.1%	24.4%	56.5%
Downward contrast	20.6 %	34.4%	45%
*Social Comparison Referents*			
Similar health problems	26.2%	22.1%	52.6%
Different health problems	33.6%	31.3%	35.1%
No health problems	37.4%	20.6%	42%
*Social Comparison Contents*			
Symptoms	2.3%	12.2%	85.5%
Mood	11.5%	22.1%	66.4%
Physical Activity	15.3%	13%	71.7%

*Likert response-scale (1 = never; 5 = very often) grouped into three categories of frequency: Low = 1–2; Medium = 3; and High = 4–5.

*Pain perception VAS* mean scores were above five points. *Anxiety* and *Depression* mean scores were 13.71 (*SD* = 4.00) and 10.73 (*SD* = 4.64), respectively.

For social comparison, 75.6% of patients used *upward contrast strategies* with high frequency, which was the most used strategy (see Table 2). In addition, 52.6% of our sample compared themselves with other *referents* with a *similar health problem* (*M* = 3.37; *SD* = 1.35; Range = 1–5) and compared *contents* such as *illness symptoms* with high frequency (85.5%) *(M* = 4.36; *SD* = 0.081; Range = 1–5) (See Tables 1, 2).

### Patient Profiles

As shown in Table 3, K-means cluster analysis and differences by ANOVA were performed with the following variables: pain perception, social comparison (strategies, referents and contents), anxiety and depression. The cluster analysis identified two groups of women. Cluster 1 includes 86 patients (65.6%) showing higher *pain perception (p* < 0.001), greater use of *upward contrast* and *downward identification strategies* (*p* < 0.001), comparison with *referents* with *different* and *similar health problems* (*p* < 0.05) and *contents* such as *illness symptoms* and *mood* (*p* < 0.05), as well as higher levels of *anxiety* and *depression* (*p* < 0.001). Cluster 2 includes 45 patients (34.3%) showing lower *pain perception* (*p* < 0.001), greater use of *upward identification* (*p* < 0.001), and *downward contrast* (*p* < 0.05), lower frequency of comparison with *referents* or *contents* (*p* < 0.05), as well as lower *anxiety* and *depression* (*p* < 0.001).

**TABLE 3:** Patient profiles: Cluster analysis and differences by ANOVA.

	Cluster 1	Cluster 2		
	(*n* = 86)	(*n* = 45)		
	*M* ± *SD*	*M* ± *SD*	F	Sig.
*Pain Perception Visual Analog Scale*				
Current pain	6.06 ± 1.15	4.36 ± 1.64	6.114	**
Last week average pain	7.22 ± 1.25	5.32 ± 1.33	8.046	**
Maximum pain last week	7.78 ± 1.02	6.02 ± 1.42	7.285	**
*Social Comparison Processes in Illness Scale Strategies*				
Upward identification	8.36 ± 2.64	11.18 ± 2.72	−5.708	**
Upward contrast	12.65 ± 1.97	8.77 ± 3.27	7.226	**
Downward identification	11.01 ± 3.43	8.00 ± 3.23	4.829	**
Downward contrast	8.55 ± 2.66	10.16 ± 3.40	−2.748	*
*Referents*				
Similar health problems	3.57 ± 1.38	3.00 ± 1.24	2.308	*
Different health problems	3.21 ± 1.26	2.52 ± 1.11	3.063	*
No health problems	3.15 ± 1.52	2.75 ± 1.43	1.450	(ns)
*Contents*				
Symptoms	4.50 ± 0.75	4.07 ± .87	2.941	*
Mood	4.09 ± 0.99	3.50 ± 1.09	3.124	*
Physical Activity	3.91 ± 1.31	4.23 ± 1.01	−1.545	(ns)
*Hospital Anxiety and Depression Scale*				
Anxiety	15.30 ± 3.54	10.70 ± 3.13	7.277	**
Depression	12.50 ± 4.04	7.48 ± 3.75	6.875	**

Pain perception, social comparison (strategies, referents, and contents) and anxiety and depression. M, mean; SD, Standard Deviation; F-Fisher with *p ≤ 05; **: *p* ≤ 001; (ns), non-significant.

Finally, we show the contingency table analysis and chi-square test in order to match patient’s profiles (Cluster 1, 2) according cut-off points fixed for the HADS (Table 4). Of the sample distribution, 76.9% of anxiety *cases* and 85.2% of depression *cases* were classified according to the HADS cut-off points (HADS – Anxiety and HADS – Depression ≥ +12) for FM in Cluster 1.

**TABLE 4:** Patient profiles: Contingency table analysis and Chi-Square Test.

	Anxiety−	Anxiety+	Depression−	Depression+
	(*n* = 27)	(*n* = 104)	(*n* = 70)	(*n* = 61)
Cluster 1. (*n* = 86)	23.1%	76.9%	50%	85.2%
Cluster 2. (*n* = 45)	76.9%	23.1%	50%	14.8%
	100%	100%	100%	100%
	*χ*^2^ = 26.934**		*χ*^2^ = 18.710**	

Cluster 1, 2 according cut-off points for the HADS. Anxiety−: score < 12; Anxiety+: score ≥ 12; Depression−: score < 12; Depression+: score ≥ 12. χ^2:^: Chi-square; **p ≤ 001.

## Discussion

This study illustrates the role of social comparison processes in FM patients. We found that *upward contrast* and *downward identification* were the strategies most used by patients with FM. They also compare themselves with others (*referents*) on “similar health problems” and on *contents* such as “symptoms.” These results coincide with another recent study on FM (Terol et al., 2007b, 2012) but differ from findings in other chronic patients (rheumatoid arthritis or cancer patients) who used *upward identification* and/or *downward contrast* more often (Blalock et al., 1990; De Vellis et al., 1990; Dibb and Yardley, 2006; Terol et al., 2007b, 2012). The findings regarding the profiles in FM patients revealed two different subgroups. One of them was a “maladaptive” profile, including women with higher levels of *pain perception*, *anxiety* and *depression* and more frequent “unfavorable” social comparison *strategies* (*upward contrast* and *downward identification*). The other group, or more “adaptive” profile, included women who showed moderate levels of *pain perception*, with a lower level of *anxiety* and *depression* and more frequent “favorable” social comparison *strategies* (*upward identification* and *downward contrast*). These profiles are consistent with other studies that have correlated these variables in the same way (Terol et al., 2012; Cabrera-Perona et al., 2017) or have identified similar groups of patients in FM (Giesecke et al., 2003; De Souza et al., 2009; Calandre et al., 2011; Keller et al., 2011; Docampo et al., 2013). Giesecke et al. (2003) proposed three profiles, one of which shows moderate *anxiety / depression* and less *pain*, while another presents a higher level of *anxiety / depression* and *pain*. Using the FIQ (FM Impact Questionnaire: Burckhardt et al., 1991) other researchers also report that *pain* and stiffness appeared in all profiles, but psychological stress (*anxiety* and *depression*) was the differentiating feature between these profiles (De Souza et al., 2009; Calandre et al., 2011). According to this, in our sample, 76.9% and 85.2%, classified as “cases” of *anxiety* and *depression*, fitted into the “maladaptive” profile (HADS ≥ +12: Cabrera et al., 2015). This leads us to turn our attention toward FM profiles, but in the context of social comparison processes and their negative emotional consequences (Bair et al., 2003).

### Research and Clinical Implications

Our results are consistent with the Identification-Contrast Model (Buunk and Ybema, 1997) applied in FM or chronic illness, where frequency of *upward contrast* and *downward identification* strategies were related to psychological distress (i.e., *anxiety* and *depression*), and poor subjective well-being, quality of life or adjustment (Buunk and Gibbons, 2006; Groothof and Scholtes, 2007; Arigo et al., 2012; Terol et al., 2014; Cabrera-Perona et al., 2017). In particular, this study provides useful information about cognitive processes in women with FM, who use different *social comparison strategies* together with other relevant “comorbidity” symptoms: *perception of pain* and *anxiety* and *depression*. Lastly, this study supports some approaches toward improving more “adaptive” profiles and useful cognitive processes: (a) identifying strategies such as *upward contrast or downward identification* in order to change them, (b) encouraging positive thought thorough the use of “favorable” comparisons strategies (*downward contrast and upward identification*), which would act as a buffer to pathologic emotions and increase a better adjustment to chronic illness (Arigo et al., 2012; Terol et al., 2014; Cabrera-Perona et al., 2017), and (c) motivating the comparison processes with *referents* or “models” that provide adaptive strategies for coping and enhancing their subjective well-being.

### Limitations

The first limitation of this study is that all the participants are female. However, FM research is generally focused on women who suffer from this chronic pain. The reason why the sample consists of only women corresponds to the justified prevalence of FM diagnosis in women, as noted: the preponderance of FM in women versus men with an approximate ratio of 9:1 (Wolfe et al., 1995; Mas et al., 2008; Katz et al., 2010). Other limitations are related to the size of the sample and selection by accessibility. Although a larger sample would be beneficial, Jager et al. (2017) consider that homogeneous convenience samples (sociodemographic or clinical factors of the general population) can be a positive alternative. In this sense, we verified that our sample features were similar to those found in other FM studies.

The cluster analysis is a cross-sectional and exploratory method. Longitudinal studies and regression analysis could further clarify the role of social comparison as an antecedent or consequence of emotional responses (i.e., anxiety and / or depression). Finally, it would be very useful to ascertain the severity of chronic symptoms, uncertainty, anxiety and depression and how they change at different stages of illness and in health settings (primary care level, FM patient associations).

## Data Availability Statement

The datasets generated for this study are available on request to the corresponding author.

## Ethics Statement

The studies involving human participants were reviewed and approved by the San Vicente del Raspeig Hospital Ethics Committe. The patients/participants provided their written informed consent to participate in this study.

## Author Contributions

All authors have contributed significantly to the article. VC and MT designed the study and protocol. MB and VC carried out the data analysis and results. MM-A, AB, and MT wrote and reviewed the original draft.

## Conflict of Interest

The authors declare that the research was conducted in the absence of any commercial or financial relationships that could be construed as a potential conflict of interest.
